# Cytotoxic and Antimicrobial Constituents from the Essential Oil of *Lippia alba* (Verbenaceae)

**DOI:** 10.3390/medicines3030022

**Published:** 2016-08-12

**Authors:** Nara O. dos Santos, Renata C. Pascon, Marcelo A. Vallim, Carlos R. Figueiredo, Marisi G. Soares, João Henrique G. Lago, Patricia Sartorelli

**Affiliations:** 1 Instituto de Ciências Ambientais, Químicas e Farmacêuticas, Universidade Federal de São Paulo, Diadema 09972-270, SP, Brazil; nara.oshiro@gmail.com (N.O.d.S.); renata.pascon@gmail.com (R.C.P.); marcelo.vallim@gmail.com (M.A.V.); 2 Disciplina de Biologia Celular, Departamento de Micro, Imuno e Parasitologia, Universidade Federal de São Paulo, Sao Paulo 04021-001, SP, Brazil; rogernty@hotmail.com; 3 Instituto de Química, Universidade Federal de Alfenas, Alfenas 37130-000, MG, Brazil; marisigs@gmail.com; 4 Centro de Ciências Naturais e Humanas, Universidade Federal do ABC, Santo Andre 09210-180, SP, Brazil

**Keywords:** *Lippia alba*, essential oil, cytotoxic activity, antimicrobial activity

## Abstract

**Backgroud:** *Lippia alba* (Verbenaceae) is a plant widely used in folk medicine to treat various diseases. The present work deals with the chemical composition of the crude essential oil extracted from leaves of *L. alba* and evaluation of its antimicrobial and cytotoxic activities. **Methods:** Leaves of *L. alba* were extracted by hydrodistillation and analyzed by gas chromatography/mass spectrometry (GC/MS) as well as by nuclear magnetic resonance (NMR) spectroscopy. Cytotoxic and antimicrobial activities of crude essential oil were evaluated in vitro using MTT and broth microdilution assays, respectively. **Results:** Chemical analysis afforded the identification of 39 substances corresponding to 99.45% of the total oil composition. Concerning the main compounds, monoterpenes nerol/geraniol and citral correspond to approximately 50% of crude oil. The cytotoxic activity of obtained essential oil against several tumor cell lines showed IC_50_ values ranging from 45 to 64 µg/mL for B16F10Nex2 (murine melanoma) and A549 (human lung adenocarcinoma). In the antimicrobial assay, was observed that all tested yeast strains, except *C. albicans*, were sensitive to crude essential oil. MIC values were two to four-folds lower than those determined to bacterial strains. **Conclusion:** Analysis of chemical composition of essential oils from leaves of *L. alba* suggested a new chemotype nerol/geraniol and citral. Based in biological evidences, a possible application for studied oil as an antifungal in medicine, as well as in agriculture, is described.

## 1. Introduction

The Verbenaceae family, with tropical and subtropical distribution, is composed of approximately 90 genera, including *Lippia* [1], with more than 200 species of herbaceous plants, small shrubs, and trees [2,3]. Most of these species have traditionally been used in the treatment of gastrointestinal and respiratory diseases [4]. Several pharmacological aspects have been ascribed to the genus *Lippia*, including antimicrobial, antifungal, antimalarial, larvicidal, antispasmodic, analgesic, anti-inflammatory, and antipyretic activities. *Lippia alba* (Mill.) N.E. Brown is native to the Americas widely distributed across the Southern United States and Northern Argentina [5]. In Brazil, this plant is popularly known as lemon balm [6,7] and have been used in folk medicine for the treatment of various diseases, such as gastric diseases, diarrhea, fever, asthma, cough, and soothing, antispasmodic, and emenagoga [2,8,9,10,11,12,13]. As other species from the same genus, phytochemicals from *L. alba* exhibited antibacterial, antifungal, antiviral, antiprotozoal, analgesic, anti-inflammatory, cytotoxic, antioxidant, and acaricidal activities [5]. Essential oils obtained from the leaves of *L. alba* were previously studied and three different chemotypes were reported: myrcene/citral, limonene/citral and limonene/carvone [14]. Zoghbi et al. [15] classified three other chemotypes: eucalyptol/limonene, limonene/carvone, and citral/germacrene-d. In addition to these volatile compounds, other monoterpenes and phenylpropanoids have been described as main metabolites in the essential oils from *L. alba*: linalool, β-caryophyllene, tagetone, myrcene, γ-terpinene, camphor, estragole, eucalyptol, camphor, limonene, and piperitone [12,13,14,15,16,17,18]. This variability has been explained due to factors such as seasons, flowering time, plant age, amount of precipitation, geographic and climatic factors [8,12,19], as well as the part of the studied plant, extraction method, soil characteristics, and the genetic variability of these plants [12,20,21,22,23]. These differences may lead to a different oil composition and, therefore, to different pharmacological effects [9,12]. As part of our continuous study with volatile oils from Brazilian species [24,25,26], the present work deals with the chemical composition of the essential oil from leaves of the Brazilian species of *L. alba*, as well as the cytotoxic and antimicrobial evaluation of crude essential oil.

## 2. Materials and Methods

### 2.1. General Experimental Procedures

^1^H and ^13^C NMR spectra of crude oil were registered, respectively, at 300 and 75 MHz into a Ultrashield 300 Advance III spectrometer (Bruker, Fremont, CA, USA) using CDCl_3_ (Aldrich, St. Louis, MO, USA) as solvent and tetramethylsilane (TMS) as internal standard. Gas chromatograms were obtained on a GC-2010 gas chromatograph (Shimadzu, Kyoto, Japan) equipped with an FID-detector and AOC-20i automatic injector (Shimadzu, Kyoto, Japan) using a RtX-5 (5% phenyl, 95% polydimethylsiloxane-30 m × 0.32 mm × 0.25 μm film thickness, Restek (Bellefonte, PA, USA)) capillary column. These analyses were performed by injecting 1.0 μL of a 1.0 mg/mL solution of volatile oil in CH_2_Cl_2_ in a split mode (1:10) employing helium as the carrier gas (1 mL/min) under the following conditions: injector and detector temperatures of 220 °C and 250 °C, respectively; oven programmed temperature from 40 to 240 °C at 3 °C/min, holding 5 min at 240 °C. The percentage compositions of the oil samples were computed by internal normalization from the flame ionization detector gas chromatography (GC-FID) peak areas without using correction for response factors. Gas chromatography coupled to low resolution electronic impact mass spectrometry (GC-LREIMS) analysis was conducted in a GC-17A chromatograph interfaced with a MS-QP-5050A mass spectrometer (Shimadzu, Kyoto, Japan). The LREIMS operating conditions were an ionization voltage of 70 eV and an ion source temperature of 230 °C with the same conditions described above. The identification of the individual compounds was performed by comparison of retention indexes (determined relatively to the retention times of a series of *n*-alkanes) and comparison of recorded mass spectra with those available in the system [27].

### 2.2. Plant Material

Leaves of *L. alba* were collected at Instituto Plantarum de Estudos da Flora in Nova Odessa city (coordinates 22°46’46” S e 47°18’49” O), São Paulo State, Brazil in 2014. Botanical identification was performed by Dr. Harri Lorenzi. The voucher of the studied species has been deposited in the Herbarium Plantarum (HPL) at number Lorenzi 1.713.

### 2.3. Essential Oil Extraction and Analysis

Fresh leaves (150 g) of *L. alba* were submitted to a steam distillation in a Clevenger-type apparatus over 5 h to afford 931 mg of the crude essential oil. The obtained oil was immediately analyzed by GC-FID and GC-LREIMS. 

### 2.4. Cell Lines

The murine melanoma cell line B16F10 was originally obtained from the Ludwig Institute for Cancer Research (São Paulo, Brazil). The melanotic B16F10Nex2 subline, characterized at the Experimental Oncology Unit (UNIFESP-Federal University of São Paulo), is characterized by low immunogenicity and moderate virulence. Human breast cancer cell line (MCF-7), human lung adenocarcinoma (A549), and human umbilical vein endothelial (HUVEC) were obtained from the Ludwig Institute for Cancer Research.

### 2.5. In Vitro Cytotoxic Activity

The essential oil extracted from leaves of *L. alba* was dissolved in dimethyl sulfoxide (DMSO) to the final concentration of 10 mg/mL, diluted in RPMI medium containing 10% fetal calf serum ranging from 100 to 0 µg/mL and incubated with 1 × 10^4^ cells in a 96-well plate. After 18 h of incubation, cell viability was measured using the Cell Proliferation Kit I (MTT) (Sigma, St. Louis, MI, USA), an MTT-based colorimetric assay [27,28]. Readings were made with a plate reader at 570 nm. All experiments were performed in triplicate.

### 2.6. Media, Antibiotics, and Growth Conditions

Yeast were cultivated on agar plates containing YPD (1% yeast extract, 2% peptone, 2% dextrose, and 2% agar) or RPMI1640 (Sigma). Gram-negative bacteria were grown in LB (0.5% yeast extract, 1% tryptone, 1% NaCl, and 2% agar) and Gram-positive bacteria were tested in BHI (Himedia, Mumbai, India). Fluconazole (Sigma) was used as the positive control for yeast and chloramphenicol (Sigma) was the positive control for bacteria. Crude essential oil from *L. alba* was diluted in DMSO or saline (0.9%) plus Tween 80 (0.5%) and spotted on 5 mm sterile filter paper [25].

### 2.7. Microorganisms Strains

The bacteria and yeast species used in this work are described in the Table 1.

### 2.8. Disk Diffusion Assay

Antimicrobial activity of essential oil from leaves of *L. alba* was evaluated using the disk diffusion method according to the Clinical and Laboratory Standards Institute (CLSI, OPAS M2-A8) with modifications. Thin agar plates were prepared with 10 mL of YPD (yeast), LB (Gram-negative) and BHI (Gram-positive) media. Three milliliters of liquid cultures were grown at 30 °C with aeration (150 rpm) overnight on YPD (yeast), LB (Gram-negative), or BHI (Gram-positive). A top agar was prepared by mixing 100 µL of each culture with 10 mL of soft agar medium for confluent plates (YPD, LB or BHI plus 1% agar) and poured on top of the thin agar (2% agar medium). Sterilized 5 mm filter paper disks were then impregnated with 20 µL of crude essential oil diluted in DMSO. The disks were placed on top of agar plates and incubated at 30 °C for 24 or 48 h depending on the microorganism. Fluconazole (1 mg) and chloramphenicol (200 µg) were used as positive controls for yeast and bacteria, respectively. Negative control was prepared by impregnating the paper disks with the same amount of DMSO used to dilute the essential oil. All tests were performed in triplicate. Tested strains were selected since they represent clinical important microbes and were previously tested in other studies conducted by our research group [25].

### 2.9. Minimum Inhibitory Concentration

Microdilution tests were conducted according to the Clinical and Laboratory Standards Institute (CLSI), OPAS1 M27-A2 for yeasts and OPAS M7-A6 for bacteria according to literature [25] with slight modifications. Briefly, minimum inhibitory concentration (MIC) values were determined using microtiter plates (96 wells) with a total volume of 100 µL. Microorganisms were cultured in test tubes filled with 3 mL medium RPMI 1640 (Sigma, St. Louis, MI, USA) for yeast and BHI (Sigma, St. Louis, MI, USA) for bacteria, overnight at 30 °C in a rotary shaker (150 rpm). The cultures were diluted and adjusted to 1–2 × 10^2^ CFU/mL, which was confirmed by viability counts on YPD and BHI plates (100 µL of diluted cells). Crude essential oil and reference standards were serial diluted (two-fold) and added to each well. A sterilization control containing medium only and growth control containing cell, DMSO (10 µL), or saline (10 µL), and Tween 80 were included as negative and positive controls, respectively. Depending on the microorganism the microtiter plates were then incubated at 30 °C for 24 or 48 h. Microorganism growth was determined by reading the absorbance at 530 nm in a plate reader Logen MT-960 (Tecan, Männedorf, Switzerland) and the minimum inhibitory concentration was considered the lowest concentration at which at least 80% of growth was inhibited. All tests were performed in triplicate. 

## 3. Results and Discussion

### 3.1. Chemical Composition of Essential Oil from L. alba

The yield of the essential oil extracted from *L. alba* was 0.21% based on the weight of the fresh leaves used in the distillation procedure. Analysis of the crude oil by gas chromatography with flame ionization detector (GC-FID) and by gas chromatography coupled to low resolution electronic impact mass spectrometry (GC-LREIMS), followed by calculation of Kovats indices, and comparison with the literature data [27] allowed the identification of 39 compounds corresponding to 99.45% (Table 2) of the crude essential oil. 

**Figure 1:**
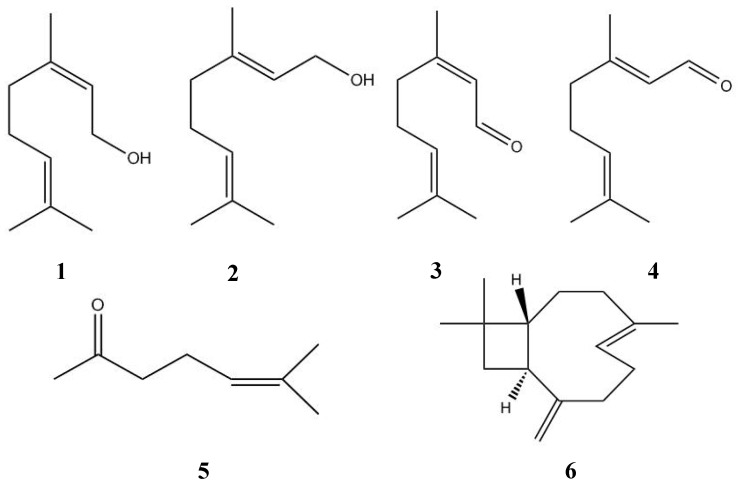
Structures of main compounds identified in essential oil from *L. alba*: nerol (**1**), geraniol (**2**), neral (**3**), geranial (**4**), 6-methy-5-hepten-2-one, (**5**) and *E*-caryophyllene (**6**).

Among the identified compounds, approximately 67% corresponds to monoterpenes and 19% to sesquiterpenes. The main characterized components were the isomeric monoterpenes nerol/geraniol (27.09%) and citral (21.87%), as well as 6-methyl-5-heptene-2-one (11.98%) and *E*-caryophyllene (9.25%), as showed in Figure 1. These compounds were previously found as minor metabolites in the essential oil from leaves of *L. alba* [29], suggesting the new chemotype nerol/geraniol and citral to the studied oil.

To confirm the identification of these main monoterpenes, the ^13^C NMR spectrum of crude oil was recorded and exhibited peaks assigned to carbonyl groups of aldehydes (C-1) at δ 191.5 and 190.9 of geranial and neral, respectively. The peaks at δ 127.4/128.6 and δ 122.5/122.2 were assigned to double bonds (C-2 and C-6), whereas those at δ 164.2/164.2 and δ 132.9/133.7 were attributed to C-3 and C-7 of geranial and neral, respectively. The secondary carbons C-4 and C-5 were represented by signals δ 40.6/25.7 and 32.6/27.0. The signals of carbons C-8 (δ 17.7) and C-9 (δ 25.6) displayed the same chemical shift for neral and geranial, but the peaks observed to C-10 were observed at δ 25.1 to neral and δ 17.6 to geranial, according to literature data [30,31]. Finally, analysis of ^1^H NMR spectrum indicated a ratio of 5:3 of geranial:neral based in the integration of the doublets at δ 9.99 (*J* = 9.0 Hz) and 9.89 (*J* = 9.0 Hz), assigned to aldehyde hydrogens (H-1).

### 3.2. Cytotoxic Activity

The essential oil from leaves of *L. alba* was evaluated for its cytotoxic activity in vitro against the lines B16F10Nex2 and A549 with IC_50_ of 45.8 µg/mL and 63.9 µg/mL, respectively, and IC_50_ higher than 100 µg/mL to the MCF-7 cell line. Comparatively, positive control cisplatin displayed IC_50_ of 52.8 µg/mL (B16F10Nex2), whereas paclitaxel showed IC_50_ of 84.3 µg/mL (A549) and 171.5 µg/mL (MCF), as showed in Table 3. Based in the obtained results, the essential oil from *L. alba* could be considered as a source of antitumor compounds, since it showed lower IC_50_ values in comparison to standard drugs in the tested cells. Additionally, the essential oil from *L. alba* was non-toxic to non-tumorigenic in HUVEC, in opposition to the cisplatin positive control, which displayed an IC_50_ value of 42.6 μg/mL.

In a previous study concerning the cytotoxic activity of essential oils from *L. alba* against cell lines CT26WT (murine colon carcinoma), A549 (human lung adenocarcinoma), MDA-MB-231 (human breast adenocarcinoma), CACO 2 (human colon carcinoma), and CHO (normal hamster ovary cells) no activity was observed to chemotype geraniol, whereas chemotype carvone inhibited A549 cells with IC_50_ of 47.80 µg/mL [32,33]. Otherwise, citral and carvone chemotypes of *L. alba* displayed dose-dependent cytotoxicity effect against HeLa (human cervical carcinoma) cells [34]. Regarding the major component citral, this compound exhibited antitumor activity. The effect of citral combined with retinoic acid showed inhibition of proliferation of A549 (lung carcinoma epithelial) in a dose dependent manner [35]. Additionally, antitumor properties was detected to micelle formulation of citral [36], whereas cytotoxicity against colon adenocarcinoma (HCT) and lung adenocarcinoma was described for citral, administrated as a mixture of monoterpenes neral + geranial and in a combination with lysine [37]. Geraniol, another main compound found in the studied essential oil from *L. alba*, was described as an inhibitor of prostate cancer growth by targeting cell cycle and apoptosis pathways [38]. 

### 3.3. Antimicrobial Activity

Initially, disk diffusion assays were conducted with the yeasts and bacterial strains listed in Table 1 in order to screen for antimicrobial activity. Four bacterial strains (two Gram-negative and two Gram-positive) were sensitive to the essential oil from *L. alba*. In sequence, the species listed in Table 4 were tested to determine the MICs. The results showed that yeasts are considerably more sensitive to essential oil from *L. alba* than bacterial strains. At 4 mg/mL only *E. coli* showed a growth inhibition over 80%; all other strains showed an MIC of 4.0 mg/mL with growth inhibitions below 70%. Conversely, all yeast strains tested were sensitive to the essential oil from *L. alba* and the MICs calculated for them was two- to four-fold lower than those found for bacterial strains. *C. dubliniensis* was the microbial strain most sensitive to essential oil from *L. alba* (0.5 mg/mL). All yeast strains tested showed, at least, 90% of growth inhibition. 

There are many reports in the literature about the antimicrobial effects of essential oils from *L. alba.* In our investigation the activity of this oil on bacterial growth was modest, suggesting that the tested crude oil does not play an important role as an antibacterial. However, future studies with purified fractions from this oil could uncover more significant biological activities against bacteria, as shown before by Klein et al. [39]. Essential oils from *L. alba* have been extensively reported as antifungal, especially against phyto [40,41,42,43] and human pathogens [43]. In summary, these results hold a promise of possible applications for *L. alba* essential oil as an antifungal in medicine, as well as in agriculture. 

## Figures and Tables

**Table 1:** Target strains used for antimicrobial activity assays.

Species	Designation
Yeast	
*Candida dubliniensis*	ATCC 7978
*Candida tropicalis*	ATCC 13803
*Candida glabrata*	ATCC 90030
*Candida parapsilosis*	Clinical isolate 68
*Candida krusei*	Clinical isolate 9602
*Candida albicans*	CBMAI * 560
*Cryptococcus grubii* (A)	ATCC 208821
*Cryptococcus gattii* (B)	ATCC MYA-4563
*Cryptococcus gattii* (C)	ATCC MYA-4560
*Cryptococcus neoformans* (D)	ATCC MYA-4567
*Saccharomyces cerevisiae*	ATCC 201389
Bacteria	
*Escherichia coli*	-
*Serratia marcescens*	CBMAI * 469
*Pseudomonas aeruginosa*	CBMAI * 602
*Staphylococcus epidermidis*	CBMAI * 604
*Enterococcus faecalis*	-

* CBMAI: Coleção Brasileira de Microrganismos do Ambiente e Indústria.

**Table 2:** Chemical composition of the essential oil obtained from leaves of *L. alba*.

Compound	IK	Relative Amount
α-pinene	939	0.15
Sabinene	976	0.41
6-methy-5-hepten-2-one	985	11.98
α-phelandrene	1005	3.25
α-terpinene	1018	0.23
β-phelandrene	1031	1.67
*Z*-β-ocimene	1040	0.18
*E*-β-ocimene	1050	2.70
γ-terpinene	1062	0.03
*cis*-sabinene hydrate	1068	0.04
Terpinolene	1088	0.02
Linalol	1098	2.14
*cis*-oxide rose	1111	0.80
Dihydrolinalol	1134	0.14
Citronelal	1153	0.45
Borneol	1165	0.33
α-terpineol	1189	1.15
Citronelol	1228	4.43
nerol/geraniol	1252	27.09
citral (neral/geranial)	1267	21.87
geranyl formate	1300	1.48
Longicyclene	1373	0.11
Longifolene	1402	1.65
α-cedrene	1409	0.62
*E*-caryophyllene	1418	9.25
*cis*-thujopsene	1429	0.12
α-humulene	1454	0.82
*allo*-aromadendrene	1461	0.25
α-acoradiene	1463	0.04
Chamigrene	1475	0.04
germacrene-D	1480	1.67
*Ar*-curcumene	1483	0.38
α-zingiberene	1495	0.97
germacrene-A	1503	0.98
β-bisabolene	1509	0.20
*Z*-γ-bisabolene	1515	0.18
*epi*-longipinanol	1561	0.03
Longipinanol	1566	1.53
(6*R*,7*R*)-bisabolone	1737	0.07
*Monoterpenes*		67.08
*Sesquiterpenes*		18.91
*Other compounds*		13.46
Total		99.45

**Table 3:** IC_50_ (μg/mL) values to the essential oil from leaves of *L. alba* and to positive control (cisplatin and paclitaxel) against cell lines.

Cell Line	IC_50_ (µg/mL)		
	Essential Oil	Cisplatin Paclitaxel	
B16F10Nex2	45.82 ± 4.16	52.8 ± 4.50	-
A549	63.98 ± 6.76	-	84.3 ± 6.83
MCF-7	>100	-	171.5 ± 16.39
HUVEC	>100	42.6 ± 2.25	-

**Table 4:** Minimum inhibitory concentrations (MIC) for essential oil from *L. alba*. Concentrations are given in mg/mL. Numbers in parentheses denote the percentage of growth inhibition and its standard deviation.

Species	Essential Oil	Positive Control
*E. coli*	4.0 (98% ± 1%)	0.01 ^a^
*S. marcescens*	4.0 (42% ± 3%)	0.01 ^a^
*E. faecalis*	4.0 (64% ± 2%)	0.02 ^a^
*S. epidermidis*	4.0 (56% ± 2%)	0.04 ^a^
*C. albicans*	2.0 (99% ± 2%)	0.025 ^b^
*C. dubliniensis*	0.5 (93% ± 2%)	0.006 ^b^
*C. tropicalis*	2.0 (99% ± 1%)	0.05 ^b^
*C. glabrata*	2.0 (99% ± 1%)	0.05 ^b^
*C. parapsilosis*	2.0 (99% ± 1%)	0.006 ^b^
*C. krusei*	2.0 (99% ± 1%)	0.05 ^b^
*C. grubii* (A)	1.0 (99% ± 2%)	0.013 ^b^
*C. gattii* (B)	2.0 (97% ± 2%)	0.025 ^b^
*C. gattii* (C)	2.0 (98% ± 1%)	0.006 ^b^
*C. neoformans* (D)	1.0 (98% ± 2%)	0.006 ^b^
*S. cerevisiae*	2.0 (99% ± 1%)	0.013 ^b^

^a^ chloramphenicol; ^b^ fluconazole.

## References

[1] Trease G.E., Evans W.C. (1983). Pharmacognosy.

[2] Pascual M.E., Slowing K., Carretero E., Mata D.S., Villar A. (2001). *Lippia*: Traditional uses, chemistry and pharmacology: A review. J. Ethnopharmacol..

[3] Terblanché F.C., Kornelius G. (1996). Essential oil constituents of the genus *Lippia* (Verbenaceae)—A literature review. J. Essent. Oil Res..

[4] Morton J.F., Charles C. (1981). Atlas of medicinal plants of middle America: Bahamas to Yucatan.

[5] Hennebelle T., Sahpaz S., Joseph H., Bailleul F. (2008). Ethnopharmacology of *Lippia alba*. J. Ethnopharmacol..

[6] Jannuzzi H., Mattos J.K.A., Silva D.B., Gracindo L.A.M., Vieira R.F. (2011). Avaliação agronômica e química de dezessete acessos de erva-cidreira (*Lippia alba* (Mill.) N.E. Brown)—Quimiotipo citral, cultivados no Distrito Federal. Rev. Bras. Plantas Med..

[7] Matos F.J.A. (2000). Plantas Medicinais: Guia de seleção e emprego de plantas usadas em fitoterapia no Nordeste do Brasil.

[8] Corrêa C.B.V. (1992). Contribuição ao estudo de *Lippia alba* (Mill.) N.E. Br. ex Britt & Wilson—Erva-cidreira. Rev. Bras. Farmacogn..

[9] Matos F.J.A. (1996). As ervas cidreiras do Nordeste do Brasil—Estudo de três quimiotipos de *Lippia alba* (Mill.) N.E. Brown (Verbenaceae). Parte II—Farmacoquímica. Rev. Bras. Farm..

[10] Gupta M. (1995). 270 Plantas Medicinales Iberoamericanas.

[11] Morais S.M., Dantas J.D.P., Silva A.R.A., Magalhães E.F. (2005). Plantas medicinais usadas pelos índios Tapebas do Ceará. Rev. Bras. Farmacogn..

[12] Tavares E.S., Julião L.S., Lopes D., Bizzo H.R., Lage C.L.S., Leitão S.G. (2005). Análise do óleo essencial de folhas de três quimiotipos de *Lippia alba* (Mill.) N.E. Br. (Verbenaceae) cultivados em condições semelhantes. Rev. Bras. Farmacogn..

[13] Pinto E.P.P., Amorozo M.C.M., Furlan A. (2006). Conhecimento popular sobre plantas medicinais em comunidades rurais de mata atlântica-Itacaré, BA, Brasil. Acta Bot. Bras..

[14] Matos F.J.A., Machado M.I.L., Craveiro A.A., Alencar J.W. (1996). Essential oil composition of two chemotypes of *Lippia alba* grown in Northeast Brazil. J. Essent. Oil Res..

[15] Zoghbi M.G.B., Andrade E.H.A., Santos A.S., Silva M.H.L., Maia J.G. (1998). Essential oils of *Lippia alba* (Mill.) N.E. Br. growing wild in the Brazilian Amazon. Flavour Fragr. J..

[16] Hennebelle T., Sahpaz S., Joseph H., Bailleul F. (2006). Phenolics and iridoids of *Lippia alba*. Nat. Prod. Commun..

[17] Dellacassa E., Soler E., Menéndez P., Moyna P. (1990). Essential oils from *Lippia alba* (Mill.) N.E. Brown and *Aloysia chamaedrifolia* Cham. (Verbenaceae) from Uruguay. Flavour Fragr. J..

[18] Senatore F., Rigano D. (2001). Essential oil of two *Lippia* spp. (Verbenaceae) growing wild in Guatemala. Flavour Fragr. J..

[19] Machado T.F., Pereira R.C.A., Batista V.C.V. (2014). Seasonal variability of the antimicrobial activity of the essential oil of *Lippia alba*. Rev. Ciênc. Agron..

[20] Alea J.A.P., Luis A.G.O., Pérez A.R., Jorge M.R., Baluja R. (1996). Composición y propriedades antibacterianas del aceite esencial de *Lippia alba* (Mill.) N.E. Brown. Rev. Cuba. Farm..

[21] Stashenko E.E., Jaramillo B.E., Martínez J.R. (2004). Comparison of different extraction methods for the analysis of volatile secondary metabolites of *Lippia alba* (Mill.) N.E. Brown, grown in Colombia, and evaluation of its in vitro antioxidant activity. J. Chromatogr. A.

[22] Castro D.M., Ming L.C., Marques M.O.M. (2002). Composição fitoquímica dos óleos essenciais de folhas da *Lippia alba* (Mill). N.E. Br. em diferentes épocas de colheita e partes do ramo. Rev. Bras. Plantas Med..

[23] Viccini L.F., Silveira R.S., Vale A.A., Campos J.M.S., Reis A.C., Santos M.O., Campos V.R., Carpanez A.G., Grazul R.M. (2014). Citral and linalool content has been correlated to DNA content in *Lippia alba* (Mill.) N.E. Brown (Verbenaceae). Ind. Crop. Prod..

[24] Bou D.D., Lago J.H.G., Figueiredo C.R., Matsuo A.L., Guadagnin R.C., Soares M.G., Sartorelli P. (2013). Cytotoxicity evaluation of essential oil, zingiberene and derivatives from leaves of *Casearia sylvestris* (Salicaceae). Molecules.

[25] Santos N.O., Mariane B., Lago J.H.G., Sartorelli P., Rosa W., Soares M.G., da Silva A.M., Lorenzi H., Vallim M.A., Pascon R.C. (2015). Assessing the chemical composition and antimicrobial activity of essential oils from Brazilian plants—*Eremanthus erythropappus* (Asteraceae), *Plectrantuns barbatus*, and *P. amboinicus* (Lamiaceae). Molecules.

[26] Grecco S.S., Martins E.G., Girola N., de Figueiredo C.R., Matsuo A.L., Soares M.G., Bertoldo B.C., Sartorelli P., Lago J.H.G. (2015). Chemical composition and in vitro cytotoxic effects of the essential oil from *Nectandra leucantha* leaves. Pharm. Biol..

[27] Adams R.P. (2007). Identification of Essential Oil Components by Gas Chromatography/Mass Spectrometry.

[28] Mosmann T. (1983). Rapid colorimetric assay for cellular growth and survival: Application to proliferation and cytotoxicity assays. J. Immunol. Meth..

[29] Soares L. (2001). Estudo tecnológico, fitoquímico e biológico de *Lippia alba* (Miller) N.E. Brown Ex Britt. & Wils. (falsa-melissa) Verbenaceae. Master’s Thesis.

[30] Glamoclija J., Sokovic M., Tesevic V., Linde G.A., Colauto N.B. (2011). Chemical characterization of *Lippia alba* essential oil: An alternative to control green molds. Braz. J. Microbiol..

[31] Ragasa C.Y., Ha H.K., Hasika M., Maridable J.B., Gaspillo P.D., Rideout J.A. (2008). Antimicrobial and Cytotoxic Terpenoids from *Cymbopogon citratus* Stapf. Philipp. Scient. J..

[32] Jeon J.H., Lee C.H., Lee H.S. (2009). Food protective effect of geraniol and its congeners against stored food mites. J. Food Prot..

[33] Gomide M.S., Lemos F.O., Lopes M.T.P., Alves T.M.A., Viccini L.F., Coelho C.M. (2013). The effect of the essential oils from five different *Lippia* species on the viability of tumor cell lines. Rev. Bras. Farmacogn..

[34] Mesa-Arango A.C., Montiel-Ramos J., Zapata B., Durán C., Betancur-Galvis L., Stashenko E. (2009). Citral and carvone chemotypes from the essential oils of Colombian *Lippia alba* (Mill.) N.E. Brown: Composition, cytotoxicity and antifungal activity. Mem. Inst. Oswaldo Cruz.

[35] Farah I.O., Trimble Q., Ndebele K., Mawson A. (2010). Retinoids and citral modulated cell viability, metabolic stability, cell cycle progression and distribution in the A549 lung carcinoma cell line. Biomed. Sci. Instrum..

[36] Zeng S., Kapur A., Patankar M.S., Xiong M.P. (2015). Formulation, Characterization, and Antitumor Properties of Trans- and Cis-Citral in the 4T 1 Breast Cancer Xenograft Mouse Model. Pharm. Res..

[37] Shi C., Zhao X., Liu Z., Meng R., Chen X., Guo N. (2016). Antimicrobial, antioxidant, and antitumor activity of epsilon-poly-l-lysine and citral, alone or in combination. Food Nutr. Res..

[38] Kim S.H., Bae H.C., Park E.J., Lee C.R., Kim B.J., Lee S., Park H.H., Kim S.J., So I., Kim T.W. (2011). Geraniol inhibits prostate cancer growth by targeting cell cycle and apoptosis pathways. Biochem. Biophys. Res. Commun..

[39] Klein G., Rüben C., Upmann M. (2013). Antimicrobial activity of essential oil components against potential food spoilage microorganisms. Curr. Microbiol..

[40] Tomazoni E.Z., Pansera M.R., Pauletti G.F., Moura S., Ribeiro R.T., Schwambach J. (2016). In vitro antifungal activity of four chemotypes of *Lippia alba* (Verbenaceae) essential oils against *Alternaria solani* (Pleosporeaceae) isolates. An. Acad. Bras. Cienc..

[41] Anaruma N.D., Schmidt F.L., Duarte M.C., Figueira G.M., Delarmelina C., Benato L.A., Sartoratto A. (2010). Control of *Colletotrichum gloeosporioides* (Penz.) Sacc. in yellow passion fruit using *Cymbopogon citratus* essential oil. Braz. J. Microbiol..

[42] Shukla R., Kumar A., Singh P., Dubey N.K. (2009). Efficacy of *Lippia alba* (Mill.) N.E. Brown essential oil and its monoterpene aldehyde constituents against fungi isolated from some edible legume seeds and aflatoxin B1 production. Int. J. Food Microbiol..

[43] Singh R.K. (2005). Fungitoxicity of some higher plants and synergistic activity of their essential oils against *Sclerotium rolfsii* Sacc. causing foot-rot disease of barley. Hind. Antibiot. Bull..

